# Synchronous Gastric and Colon Cancer

**DOI:** 10.7759/cureus.48437

**Published:** 2023-11-07

**Authors:** Nicolas Campuzano, Tatiana Fernandez Trokhimtchouk, Luis F Flores, Estefanie S Otanez, Edwin Guallasamín

**Affiliations:** 1 General Surgery, Universidad Internacional del Ecuador, Quito, ECU; 2 Surgical Oncology, Hospital Oncológico Solón Espinosa Ayala (SOLCA), Quito, ECU

**Keywords:** colectomy, gastrectomy, immunohistochemistry, metachronous cancer, synchronous cancer, colon cancer, gastric cancer

## Abstract

Colorectal cancer (CRC) and gastric cancer, ranking as the third and fifth most prevalent global cancers, respectively, have seen increased diagnoses due to advancements in early detection and extended lifespans. Synchronous and metachronous cancers, with a rare incidence, are notable, with CRC being the predominant synchronous occurrence in gastric cancer patients. Screening CRC patients for gastric cancer is debated due to its low incidence, underscoring the crucial role of early diagnosis. Distinguishing between metastatic adenocarcinoma and synchronous tumors is challenging, relying on techniques such as immunohistochemistry. Surgery is the primary treatment for synchronous cancer, with successful single-stage surgeries reported. A case presentation of a 68-year-old female highlights these complexities. The final diagnosis encompassed stage I gastric cancer and stage IV colon cancer, leading to adjuvant chemotherapy. Synchronous gastric cancer and CRC present a unique clinical challenge, necessitating tailored approaches. Collaboration between surgical and oncological teams is crucial for comprehensive treatment planning and optimizing patient outcomes.

## Introduction

In the realm of global cancer diagnoses, colorectal cancer (CRC) stands as the third most frequently encountered, with gastric cancer trailing closely behind in fifth place [1]. Thanks to progress in early detection methods and the extension of human lifespans, there has been a notable surge in the identification of synchronous and metachronous cancers, with a reported incidence that ranges from 2.5% to 3.4%. Notably, CRC emerges as the predominant synchronous and metachronic occurrence in patients with gastric cancer, followed by lung and hepatic malignancies [2-4].

Given these correlations, it may seem prudent to screen CRC patients for gastric cancer. However, owing to its low incidence, no specific recommendations have been put forth. It is worth emphasizing that early diagnosis remains a pivotal determinant for enhancing the prognosis of cancer patients. This is particularly true for synchronous cancers, as timely identification enables more effective treatment strategies, ultimately leading to higher rates of remission and a reduction in both morbidity and mortality [5,6].

Distinguishing between metastatic adenocarcinoma and a synchronous tumor poses a common challenge. Several techniques are available to facilitate this diagnostic process, including immunohistochemistry. In cases of colorectal adenocarcinoma, a characteristic pattern emerges with negative cytokeratin (CK) 7 and positive CK 20 staining. Furthermore, CRC exhibits the expression of the nuclear transcription factor CDX2, which demonstrates high specificity for intestinal epithelial cells [7].

In cases where clinically feasible, surgery stands as the optimal treatment avenue for attaining disease-free survival in synchronous cancer. Numerous case reports showcase successful outcomes in both open and minimally invasive single-stage surgeries [8].

## Case presentation

A 68-year-old female with a history of arterial hypertension presented for external consultation with a three-month history of hyporexia, unintentional weight loss of 7 kilograms, occasional moderate diffuse abdominal pain, episodic distension, and ribbon-like stools. Upon physical examination, a body mass index of 20 kg/m2 was noted, along with generalized paleness, and mild tenderness in a non-distended abdomen upon palpation. Blood work revealed microcytic hypochromic moderate anemia.

**Figure 1:**
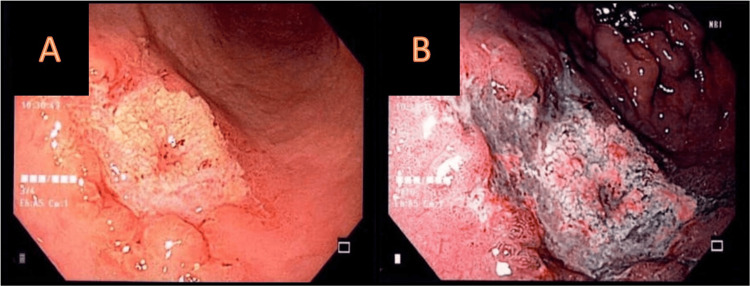
(A) An 18-mm depressed lesion with irregular elevated borders, situated 5 cm away from the cardias on the greater curvature of the stomach. (B) A deep ulcerated lesion measuring 12-mm with regular borders in the prepyloric region toward the lesser curvature.

An esophagogastroduodenoscopy revealed an 18-mm lesion on the greater curvature of the stomach, as shown in panel A of Figure 1. The pathology report confirmed moderately differentiated adenocarcinoma. Additionally, a 12-mm lesion was found in the prepyloric region, as seen in panel B of Figure 1. Pathology reported a moderately differentiated adenocarcinoma with a mucinous component.

**Figure 2:**
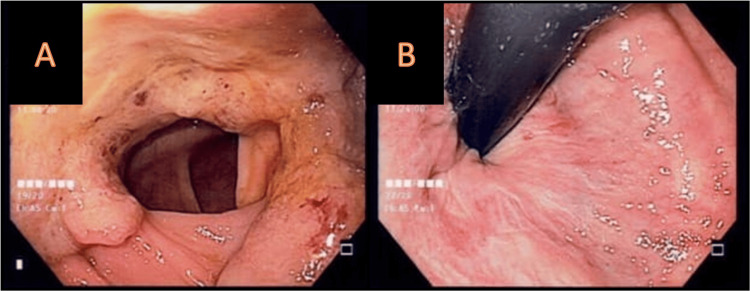
(A) A 3-cm non-stenosing deep ulcerated lesion in the ascending colon with elevated borders, comprising 75% of the circumference. (B) An ulcerated lesion encompassing 100% of the circumference, causing 80% stenosis of the lumen, preventing the passage of the endoscope, at the level of the cecum.

A subsequent colonoscopy revealed two lesions, as shown in Figure 2. Pathology of the first lesion reported a moderately differentiated adenocarcinoma with mucinous component, and pathology of the second lesion reported a well-differentiated tubular ulcerated adenocarcinoma.

**Figure 3:**
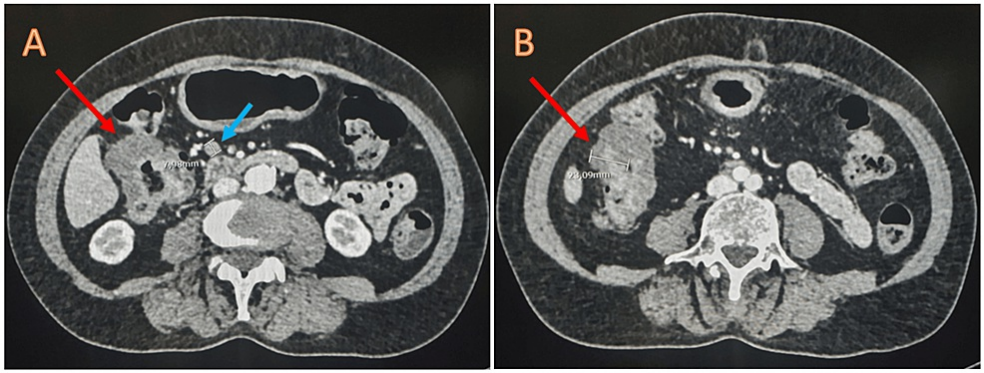
Contrast-enhanced abdominal CT (axial view). (A) The concentric thickening of hepatic flexure (red arrow), occluding 70% of the lumen, can be seen. The light blue arrow shows an 8-mm paracolic lymph node. (B) The thickest zone measured 23 mm (red arrow) at the ascending colon, obstructing 90% of the bowel lumen.

A contrast-enhanced computed tomography (CT) scan demonstrated a concentric thickening of the bowel wall at the ileocecal valve, ascending colon up to the hepatic flexure, and proximal third of the transverse colon. The thickening exhibited heterogeneous contrast enhancement, with a compromise of 70% to 90% of the lumen, accompanied by surrounding fat stranding. Several paracolic lymph nodes were identified, with the largest one measuring 8 mm (Figure 3).

**Figure 4:**
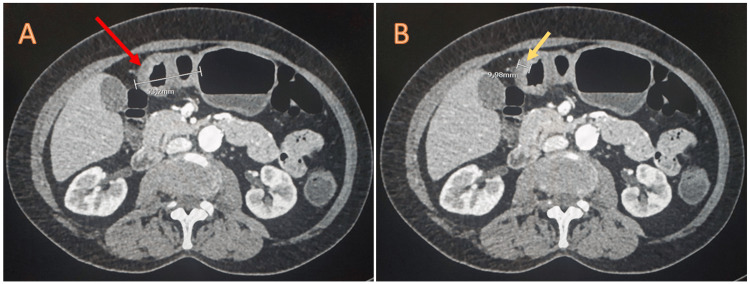
Contrast-enhanced abdominal CT (axial view) showing the gastric lesion. (A) The lesion measuring 5.3 cm. (B) Wall thickening measuring 10 mm.

The stomach displayed regular walls and was distended. Toward the antrum, a concentric thickening with contrast enhancement was observed, resulting in 40% lumen obliteration. No fat stranding or signs of metastatic tumoral activity were evident (Figure 4).

Immunohistochemistry confirmed the diagnosis of double primary tumors; the multidisciplinary team meeting concluded with a recommendation for surgical resection.

**Figure 5:**
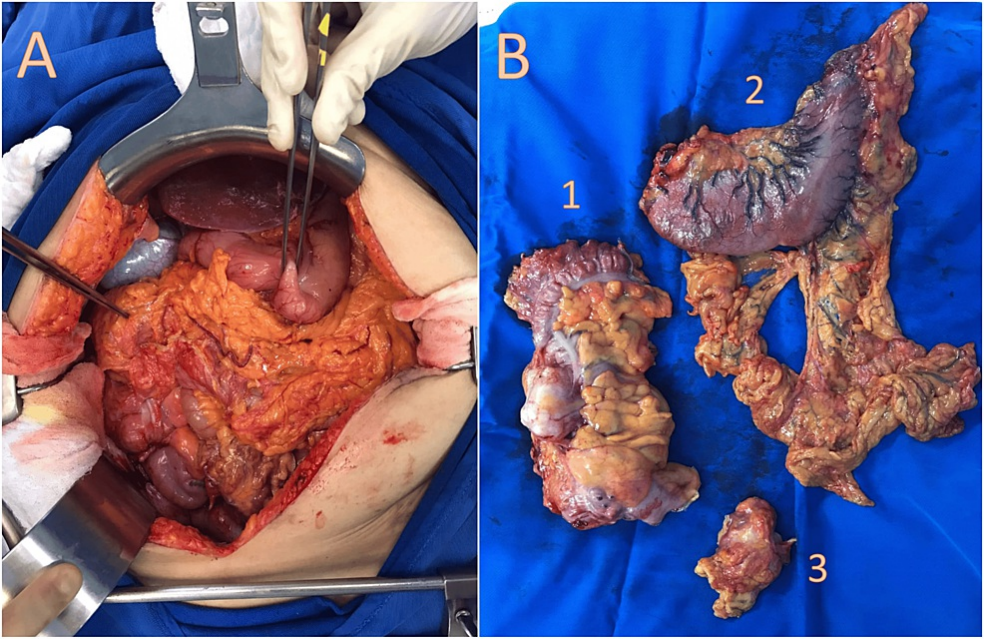
(A) An operative view of the laparotomy; the forceps are grasping one of the lesions found in the stomach. (B) The retrieved specimens: ascending and proximal third of transverse colon (1), stomach with omentum (2), and tumoral implant from the omentum (3).

During a midline laparotomy, multiple gastric tumors were discovered on the greater curvature, antrum, and prepyloric region, ranging in size from 1 to 2 cm. These tumors were mobile and showed no invasion into neighboring structures. Additionally, various tumors were found on the ascending colon, with the largest measuring 10 cm and displaying no invasion. Furthermore, a 6-cm mass, suggestive of a tumoral implant, was located on the omentum adherent to the anterior abdominal wall and subsequently resected. No free liquid or signs of carcinomatosis were observed. A right hemicolectomy with primary ileo-transverse anastomosis was performed, along with a D2 total gastrectomy with esophagojejunal anastomosis (Figure 5). Two closed-suction surgical drains were left in place, one next to the esophagojejunal anastomosis and another in the pelvis.

The postoperative period proceeded without complications, with a liquid diet initiated on postoperative day 1 and progressed according to tolerance. The patient was discharged on the sixth day after drain removal.

**Figure 6:**
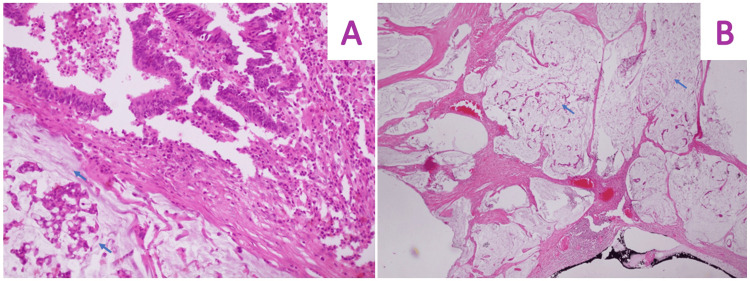
Histologic images of colonic adenocarcinoma. (A) 20x magnification. (B) 5x magnification. Strips of tumor cells in mucin lakes and the presence of signet ring cells can be seen.

The final pathology report indicated a 5 x 4 x 4 cm mucinous moderately differentiated adenocarcinoma of the ascending colon with signet ring cells (Figure 6). Invasion into the visceral peritoneum was noted, but no lymphatic invasion was reported. Margins were tumor-free, and 17 lymph nodes were isolated, with one showing positivity for malignancy. The omental implant also tested positive for malignancy. Pathological staging was determined as pT4a, pN1a, pM1a. Immunohistochemistry yielded positive results for CK20 and CDX2, and negative for CK7.

**Figure 7:**
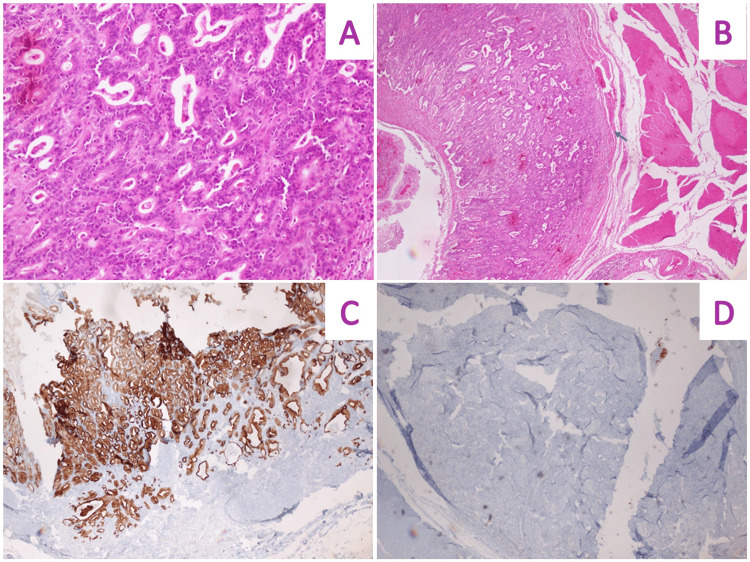
Microscopic images of gastric adenocarcinoma. (A) 20x magnification. (B) 5x magnification. Tubular adenocarcinoma infiltrating the submucosa can be seen. (C) Strongly positive staining of CK7. (D) Negative CK20 staining.

The gastrectomy report documented three tumors measuring 1.5 x 1 cm, 1.5 x 1.2 cm, and 1 x 0.7 cm in size. These were classified as well-differentiated tubular adenocarcinomas located at the body of the stomach, comprising the greater curvature, antrum, and lesser curvature at the prepyloric level. One of the lesions extended into the submucosa, exhibiting lymphovascular invasion. Immunohistochemistry was positive for CK7 and CDX2, and negative for CK20 (Figure 7). A total of 25 lymph nodes were isolated, none of which showed malignancy. Surgical margins were tumor-free. The stage was determined as mpT1b, pN0.

The final diagnosis encompassed stage I gastric cancer and stage IV colon cancer. The patient was subsequently referred for adjuvant chemotherapy, where clinical oncology started the FOLFOX (leucovorin calcium [folinic acid], fluorouracil, and oxaliplatin) regimen.

## Discussion

Synchronous cancer refers to any cancer that arises within six months of the diagnosis of another primary cancer, while metachronous cancer manifests after this time frame [5]. Some researchers posit that patients diagnosed with gastric cancer face an elevated risk of developing synchronous or metachronous CRC, suggesting a potential genetic and environmental association between these two conditions [4]. For individuals with colon cancer, it is recommended to closely monitor those at a high risk of early detection of gastric cancer. This subgroup includes males of advanced age, particularly those with a family history of solid colon tumors or a loss of MSH2 expression [3].

Synchronous gastric cancer and CRC present a unique challenge in clinical management due to their rarity and the absence of established guidelines. Unlike colon cancer, which is primarily treated with surgical resection followed by adjuvant chemotherapy as needed, gastric cancer often necessitates neoadjuvant chemotherapy as a crucial component of its treatment regimen [9].

Distinguishing between metastatic adenocarcinoma and a synchronous tumor is a frequently encountered challenge, carrying significant implications for treatment decisions and prognosis. The presence of multiple primary neoplasms adds complexity to the diagnostic process and can sometimes be mistaken for metastatic disease. Various tools in the diagnostic toolkit, including endoscopic and imaging techniques, play a crucial role in assessing patients and their familial predispositions. However, it is important to note that a conclusive diagnosis can only be reached through biopsy of any suspicious lesions [6].

Immunohistochemistry is a pivotal diagnostic tool for identifying cancer and determining tumor types. Assessing cytokeratin expression is of particular significance; there are 20 subtypes known to be expressed in various cell types and tumors. The CDX2 gene plays a crucial role in the proliferation and differentiation of intestinal epithelial cells. Studies have confirmed that the phenotype CK7-/CK20+ along with CDX2 expression serve as highly specific and sensitive markers for CRC [7].

The management of this presentation traditionally involved open surgery, necessitating extensive incisions. However, contemporary literature also highlights minimally invasive approaches. Both laparoscopic and robotic techniques have been associated with reduced postoperative pain and shorter hospital stays [10]. It is worth noting, though, that compared to open surgery, there is an increase in anastomotic leaks attributed to the lack of reinforcement of suture lines in these minimally invasive procedures [11].

In cases like the one presented, where there is uncertainty regarding which tumor is the primary and if one is a metastasis of the other or if they are truly synchronous, surgical resection remains the cornerstone of treatment. However, the optimal sequence of colectomy versus gastrectomy is still a subject of debate. As a general practice, initiating with gastrectomy has been suggested [12]. 

Additionally, the choice of incision for specimen retrieval is a critical consideration. Utilizing a Pfannenstiel incision is recommended, as it has been associated with a lower risk of ventral hernia and surgical site infection, and yields a more favorable aesthetic outcome [13].

## Conclusions

In conclusion, synchronous gastric cancer and CRC pose a unique clinical challenge. The rarity of these cases and the absence of established guidelines necessitate a tailored approach based on careful consideration of individual patient factors. Close collaboration between surgical and oncological teams is essential to formulate a comprehensive treatment plan and optimize patient outcomes.
